# 肺癌发生发展的遗传多样性选择假说——分子进化与临床意义

**DOI:** 10.3779/j.issn.1009-3419.2023.101.34

**Published:** 2023-12-20

**Authors:** Baodong LIU

**Affiliations:** 100053 北京，首都医科大学宣武医院胸外科; Department of Thoracic Surgery, Xuanwu Hospital, Capital Medical University, Beijing 100053, China

**Keywords:** 肺肿瘤, 遗传多样性, 选择, 分子进化, 临床意义, Lung neoplasms, Genetic diversity, Selection, Molecular evolution, Clinical significance

## Abstract

到目前为止，阐述肿瘤发生发展的单克隆假说不能自圆其说。遗传多样性选择假说将孟德尔遗传学与达尔文进化论联系起来，认为多克隆起源-单克隆选择-亚克隆扩张的肿瘤细胞群遗传多样性是选择压力的结果。正常细胞获得致癌驱动基因突变，相对于其他细胞具有选择优势，成为肿瘤启动细胞；在与肿瘤微环境（tumor microenvironment, TME）的互动中，绝大多数启动细胞会被人体自身免疫系统识别并杀灭，如果发生免疫逃逸，恶性肿瘤的发生率会极大增加，并发生亚克隆扩张、肿瘤内异质性等。本文提出遗传多样性选择假说，并分析其临床意义。

## Abstract (en)

So far, the monoclonal hypothesis of tumor occurrence and development cannot be justified. The genetic diversity selection hypothesis for the occurrence and development of lung cancer links Mendelian genetics with Darwin's theory of evolution, suggesting that the genetic diversity of tumor cell populations with polyclonal origins-monoclonal selection-subclonal expansion is the result of selection pressure. Normal cells acquire mutations in oncogenic driver genes and have a selective advantage over other cells, becoming tumor initiating cells; In the interaction with the tumor microenvironment (TME), the vast majority of initiating cells are recognized and killed by the human immune system. If immune escape occurs, the incidence of malignant tumors will greatly increase, and subclonal expansion, intratumour heterogeneity, etc. will occur. This article proposed the hypothesis of genetic diversity selection and analyzed its clinical significance.

2015年，Tomasetti和Vogelstein^[1]^发现人体癌症风险只有1/3归因于环境因素或遗传，2/3是由于“运气不好”，即正常干细胞的DNA复制错误。他们的研究被错误地解读为大多数癌症既不是遗传也不是环境因素造成的，只是“运气不好”^[2]^。然而，Hannun团队^[3]^研究发现，DNA复制错误对大部分癌症的发生影响甚微（低于10%-30%），60%-90%的癌症危险因素是由环境因素引起的，因此是可以改变的。Tomasetti和Vogelstein^[4]^为进一步证明自己的假说，又从全球涵盖48亿人口的69个国家收集数据，对17种癌症的正常干细胞分裂次数进行分析，结果表明大部分癌症发生的原因不是环境因素和遗传，而真的是因为“运气不好”，许多不同组织类型癌症的终生风险与正常干细胞分裂总次数的对数相关系数密切相关。就肺癌的发生而言，环境因素占66.1%，遗传占0.5%，细胞分裂占33.4%，但是，到目前为止，阐述肿瘤发生发展的单克隆起源Nowell假说不能自圆其说^[5]^，为此本文提出遗传多样性选择假说，并分析其临床意义。

## 1 遗传多样性选择假说

人类基因组中约有30亿个碱基对，每次细胞分裂通过DNA复制将遗传信息传递给子代细胞，利用DNA指导RNA合成，通过RNA指导蛋白质的合成。基于生物学大数据的分析指出，在DNA复制过程中会随机产生碱基突变并传递给子代细胞，在每次复制过程中平均会产生3个复制错误^[4]^；这种复制错误是不可避免的，是人类保持基因多样性以及持续进化的必然要求，也是成癌的必要条件。DNA复制错误包括自发脱氨、载脂蛋白B mRNA编辑酶催化多肽（apolioprotein B mRNA-editing enzyme catalytic polypeptide, APOBEC）介导编辑、DNA修复缺陷和活性氧损伤等。大部分DNA复制错误对细胞的正常功能是有害的，机体的修复酶可以持续识别和修复出错的DNA。即使发生原癌基因激活、抑癌基因缺失等驱动基因突变，由于机体进化出多种机制来阻遏突变细胞成癌，大多数突变会被及时修复，因此极少发生癌症。没有被修复的突变一般不会影响生物功能，只有极少突变会改变影响细胞功能的关键分子的活性，导致细胞恶性增殖。通过遗传分析突变特征发现，这些突变可能是遗传的，也可能是由环境因素引起的，或者是来自生殖细胞（或遗传突变，占5%-10%）和体细胞的复制突变（占90%以上）。环境因素和免疫监视等诸多因素通过影响肿瘤细胞的分裂次数、表观遗传学、染色体不稳定性（chromosomal instability, CIN）或拷贝数异常（copy number variantion, CNV）等形成优势选择和克隆清除等导致肿瘤的发生发展。

肺癌发生发展的遗传多样性选择假说将孟德尔遗传学与达尔文进化论联系起来，认为多克隆起源-单克隆选择-亚克隆扩张的肿瘤细胞群遗传多样性是选择压力的结果。正常细胞获得致癌驱动基因突变，相对于其他细胞获得选择优势，成为肿瘤启动细胞；在与周围正常细胞或肿瘤微环境（tumor microenvironment, TME）的互动中，通过选择和适应，绝大多数启动细胞会被周围正常细胞或人体自身免疫系统识别并杀灭。如果发生晚期突变、代谢重排、表观遗传重塑等，生存竞争邻近细胞形成早期癌症克隆建立，诱导TME形成，通过免疫逃逸机制发生恶性肿瘤的几率明显增加。因此，肿瘤的发生发展是内外环境因素选择压力和机体相互适应的结果。

## 2 外源性环境因素

宿主的外环境（烟草、空气污染、感染和辐射暴露）可以直接引起致癌驱动基因突变，或者通过引起组织细胞损伤的修复过程增加细胞分裂次数，从而间接增加致癌驱动基因突变的概率。外源性环境因素一方面作为致癌物质引起多种多样的不确定变异，另一方面又按照比较稳定的标准不断筛选与淘汰部分变异，并赋予已经携带致癌驱动基因突变的选择优势，最终克服阻止肿瘤启动细胞扩增的力量，从而逐步诱导易感细胞谱系中的肿瘤发生。

研究^[6,7]^表明，87%的肺癌与吸烟（包括被动吸烟）有关，吸烟会使肺癌的风险增加10-30倍；二手烟也是公认的肺癌危险因素（风险增加20%-30%），与暴露程度呈正相关。持续吸烟的人到75岁时罹患肺癌的累计风险为14%-16%（相当于每6-7个抽烟的人中就有1人在75岁前罹患肺癌），持续重度吸烟者的风险为20%-25%（相当于每4-5人中就有1人罹患肺癌）。40岁戒烟可降低80%的肺癌发病风险；50岁戒烟可降低60%左右。在中国，2003年吸烟者比例为26%（95%CI: 25.8%-26.2%），2008年为24.9%（95%CI: 24.8%-25.1%），2013年为25.2%（95%CI: 25.1%-25.4%）。中国肺癌负担与高吸烟率直接相关，特别是在男性；中国女性的肺癌发病率正在上升，这可能与空气污染或二手烟有关。其中暴露于煤炭使用者的风险特别高（OR=4.93, 95%CI: 3.73-6.52）。在中国，戒烟时间越长，罹患肺癌风险越低，戒烟时间为10年OR值为7.16（95%CI: 4.70-10.91），10-20年OR值为2.12（95%CI: 1.16-3.86），20年以上OR值为1.47（95%CI: 0.37-3.20）^[8]^。

Dong等^[9]^利用单细胞多位移扩增的方法，对14例年龄在11-86岁的不吸烟者和19例44-81岁的吸烟者的近端支气管基底细胞（proximal bronchial basal cells, PBBCs）进行了全基因组体细胞突变谱分析。单细胞中单核苷酸变异（single nucleotide variants, SNVs）的分析结果显示每个细胞每年约发生28个突变；另外，小片段插入和缺失（insertion and deletions, INDELs）的中位数也被发现随着年龄的增长而增长，每年每个细胞大约有2个INDELs，但是缺乏统计学意义。与不吸烟者相似，吸烟者细胞中的突变频率同样随着年龄的增长而增加，并且增加速度显著提高，每年每个细胞约发生91个SNVs。吸烟者PBBCs的单碱基突变（single-base substitution, SBS）特征分析显示了SBS4的存在，而SBS4在从不吸烟者中几乎是不存在的。该研究还发现：在肺细胞中检测到的细胞突变数量随着吸烟年数的延长而直线增加，但有趣的是，细胞突变的增加在暴露了23包年（每天吸烟的包数×吸烟年数）后达峰，提示DNA复制突变与机体修复损伤能力的再平衡。

## 3 遗传易感性

在国际肺癌联盟的一项大型病例对照研究^[10]^中，在调整吸烟和其他混杂因素后，肺癌患者一级亲属的肺癌风险增加了1.51倍。在纳入84项研究进行的meta分析^[11]^中，约8%的肺癌病例与遗传易感性有关，合并肺癌家族史的家族成员患肺癌风险相比其他人群高1.85倍，亚洲人群的家族聚集倾向似乎比西方人群更明显（2.14倍 vs 1.73倍），只有1个亲属患肺癌，则风险高1.55倍；有2个及以上亲属罹患肺癌，则风险高2.72倍。

## 4 内源性环境因素

### 4.1 TME

TME是由肿瘤细胞、浸润性免疫细胞、肿瘤相关成纤维细胞、血管内皮细胞以及这些细胞分泌的因子和细胞外基质非细胞成分组成的动态复杂生态系统，它们的相互作用共同决定了肿瘤的发生发展以及对治疗的反应。一方面，肿瘤细胞通过分泌多种分子作用于间质细胞等使其重编程，降低免疫原性或发挥免疫抑制而发生免疫逃逸；另一方面，TME中的间质细胞也对肿瘤克隆新抗原通过正负选择产生选择优势或选择性清除^[12]^。

在成癌早期，肿瘤细胞会采用多种手段逃避免疫攻击，例如人类白细胞抗原（human leukocyte antigen, HLA）-I类基因缺失导致的肿瘤新抗原提呈功能下调机制或诱导抑制性免疫检查点分子如抗细胞程序性死亡配体1（programmed cell death ligand 1, PD-L1）表达；同时，肿瘤细胞会劫持免疫细胞，如中性粒细胞、M2巨噬细胞和调节性T细胞（regulatory T cells, Tregs），协调免疫抑制性的TME；肿瘤细胞会模仿正常细胞分泌一些因子，如转化生长因子β（transforming growth factor β, TGF-β），欺骗免疫细胞对它的检查。当肿瘤细胞发展到一定数量的时候，它就会对免疫细胞发动攻击。近年来，单细胞RNA测序（single cell RNA sequencing, scRNA-seq）和空间转录组分析为了解TME的异质性提供了技术支持。

### 4.2 肺微生物组

在过去的10年中，随着免培养细菌鉴定技术的发展，研究人员对微生物组如何影响疾病的了解呈指数级增长，并突出了将其用作包括恶性肿瘤在内的许多疾病的诊断生物标志物和干预靶点的潜在机会。前期的研究集中在粪便微生物组上，已知肺部有一层薄薄的抑菌液体，是微生物的低营养环境，大多数肺微生物组属于拟杆菌门、厚壁菌门、变形菌门和放线菌门，每平方厘米大约有2.2×10^3^个细菌基因组，而肠道中的微生物群落为3×10^13^个。下呼吸道是肠道和上呼吸道微生物直接双向迁移和消除以维持动态细菌种群的栖息地，通常称为肠-肺轴；细胞因子和免疫细胞可以通过黏膜淋巴管和体循环在肠-肺之间移动，控制共生细菌的丰度和多样性，防止潜在致病菌群的过度生长，调节黏膜免疫和维持免疫耐受与炎症之间的平衡。在肺癌的动物模型和小型患者队列研究中，微生物群失调不仅可能影响肿瘤进展和对治疗（尤其是免疫治疗）的反应，而且还通过影响早期致癌途径在癌症发病机制中发挥关键作用^[13]^。

## 5 肿瘤发生

### 5.1 基因组异常

广义的基因组异常包括基因组不稳定性和CIN。前者包括点突变如SNVs、INDELs、基因组扩增和重排、体细胞CNV和结构变异（structure variantions, SV）；后者包括染色体碎裂、全基因组加倍（whole genome doubling, WGD）、染色体异倍性、杂合性缺失（loss of heterozygosity, LOH）、染色体重排、染色体片段缺失或扩增、染色体插入和倒位等。只有小部分驱动基因突变在癌变过程中发挥作用，这些突变在正常和肿瘤组织中具有显著不同的突变频率或者选择优势；抑癌基因的多突变与双等位缺失可能具有更大的促癌效力；染色体CNV和更大规模的SV也是肿瘤分子进化过程的重要特征。

### 5.2 表观遗传异常

表观遗传异常主要表现为基因型不变而表型发生改变，包括DNA甲基化、组蛋白甲基化、染色质重塑和各类非编码RNA调控异常。与基因组异常类似，表观遗传异常在正常组织和器官中也并不罕见，尤其是在衰老的组织细胞中，这种现象也被称为表观遗传漂移。表观遗传异常通常发生在基因组变异之前，为恶性细胞提供变异的土壤；或者发生在已经恶变的细胞克隆内，属于相对晚期的事件^[14]^。

### 5.3 肿瘤内异质性（intratumour heterogeneity, ITH）

ITH是指肿瘤形成过程中，克隆分化产生多个不同的亚克隆，导致肿瘤细胞在时间和空间上发生的异质性改变。20世纪90年代末的微阵列技术和2005年第二代测序技术（next-generation sequencing, NGS）的诞生改变了该领域的研究范式，但是标准NGS方法对提供关于肿瘤亚克隆结构的信息有限，由此开发了深度测序、多区域测序和单细胞DNA测序技术。

## 6 分子进化

癌症通常遵循分子进化模式发生发展。癌症克隆进化动力学研究的核心范式涉及体细胞在时间和空间进化模式的系统发育树，包括复制、可遗传变异、遗传漂移、选择和环境变化^[15]^。1976年，Nowell^[5]^首先提出单克隆进化的概念，接下来的几十年的研究^[16]^表明，肿瘤细胞的发生发展并不总是随机的，选择压力决定了肿瘤和克隆谱系的进化轨迹，可通过突变频率（1/f^2^）、突变丰度、变异等位基因频率（variant allel frequency, VAF）、非同义突变/同义突变比率（dN/dS）、选择压力等理解肿瘤的分子进化。大多数分子进化研究都是从一个时间点推断样本进化史，一般通过ITH构建系统发育树推论肿瘤克隆谱系进化顺序。

分子进化模型分为线性进化（linear evolution, LE）、分支进化（branching evolution, BE）、平行进化（parallel evolution）、趋同进化（convergent evolution）、中性进化（neutral evolution, NE）和间断进化（punctuated evolution, PE）等。LE及BE模型主要体现基因组的SNVs及INDELs，LE模型缺少中间分类群且具有选择优势，BE模型有中间分类群；PE模型主要体现CNV及SV，缺乏中间分类群；NE模型不具有优势选择或适应度变化，有中间分类群^[17⇓-19]^。

## 7 临床意义

### 7.1 肺腺癌发生

无驱动基因激活的肺结节更多地表现为非典型腺瘤样增生（atypical adenomatous hyperplasia, AAH）以及原位腺癌（adenocarcinoma in situ, AIS）；AAH和AIS在无驱动基因群体中占比46.2%，出现表皮生长因子受体（epidermal growth factor receptor, EGFR）突变概率为12.8%^[20]^。微浸润腺癌（minimally invasive adenocarcinoma, MIA）的EGFR突变率为10.0%；而浸润性腺癌（invasive adenocarcinoma, IAC）的EGFR突变率为28.8%^[21]^。肺腺癌的发生经历3个阶段^[22]^：第一阶段：吸烟等内外环境因素诱导的KRAS或BRAF异常激活，促使正常细胞向AAH/AIS转化；第二阶段：在EGFR突变驱动下，AAH/AIS向MIA转化；第三阶段：包括在EGFR、KRAS以及TP53的突变驱动下，进一步转变为IAC。

肺癌驱动基因突变通常与导致信号蛋白结构性激活的事件有关，这种事件通常发生在受体酪氨酸激酶（receptor tyrosine kinase, RTK）/RAS/RAF通路的癌基因中^[23]^。KRAS突变与早期肺癌预后不良高度相关；EGFR家族参与细胞运动、血管生成、细胞增殖、凋亡，与改善预后有关；BRAF位于RAS蛋白下游，在RAS-MAPK途径中起着至关重要的作用，与预后无关；MAP2K1编码一种作用于BRAF下游的蛋白。EGFR和BRAF中的驱动基因突变已被证明是独特的；此外，EGFR和KRAS中的不同突变特征已被证明在很大程度上是相互排斥的，并与不同的人口群体有关。驱动基因如HER2扩增和MET跳跃突变可能参与激活RTK/RAS/RAF通路。

及EGFR、RAS和RB1的突变一般发生在进化早期，而包括SMAD4等在内的更多癌基因的突变容易发生在进化晚期。

TRACERx 421队列中的805个原发肿瘤区域和来自248个肺腺癌的121对转移样本的全外显子组测序数据，以及来自463个原发肿瘤区域的RNA测序数据，与详细的全肿瘤和局部组织病理学分析相结合。以高级别成分为主的肿瘤表现出染色体复杂性增加，LOH和亚克隆体细胞CNV的负担更高。以高级别成分为主的肿瘤的个别区域表现出较高的增殖性和较低的克隆多样性，可能反映了近期大量的亚克隆扩张。3p和3q染色体的主干缺失在低/中级别为主的肿瘤中增加，而纯未分化的实体型肿瘤与具有实体成分的混合模式肿瘤相比，具有更高的主干臂或局灶3q重复和SMARCA4基因改变的频率，表明不同的进化轨迹^[24]^。

Jamal-Hanjani等^[25]^通过多区域全外显子组测序对100例肺癌追踪进化过程，发现肺癌患者广泛存在驱动基因突变和CNV，这些基因包括EGFR、MET、BRAF和TP53等；进化后期驱动基因突变超过肿瘤的75%，主要涉及染色质修饰及DNA损伤反应和修复（PIK3CA和NF1）；染色体不稳定直接促进了肿瘤异质性，导致了驱动基因CNV的平行进化，包括CDK4、FOXA1和BCL11A扩增。

### 7.2 多原发肺癌（multiple primary lung cancer, MPLC）

MPLC的病因起源于“场癌变效应”（Field Cancerization Effect）的概念，认为整个呼吸系统长期暴露于致癌因素中，致使支气管肺泡上皮广泛异型增生癌变，具有空间和时间的异质性^[26]^。

有研究^[27]^发现，在队列1中，EGFR、KRAS、BRAF和ALK 突变的一致率为96%。在队列2中，同一肺叶多个结节，36%为MPLC，40%为肺内转移瘤，24%的分子检测未发现有用的信息。对于多个肺叶结节的患者，81.6%为MPLC，7.4%为肺内转移瘤，11%的分子检测未发现有用的信息。在多发肺结节样肺癌的治疗上，通过切除所谓主病灶的基因检测结果使用相关靶向药物治疗其他结节是没有依据的^[28]^。

### 7.3 原发与转移

绝大多数癌症相关死亡（约90%）是由转移而非原发性肿瘤引起的。越来越多的证据^[29,30]^表明，将转移过程视为一个由物理途径控制的过程过于简单。肿瘤细胞在原发部位、转移路径、转移部位之间有复杂的动态流动，表现为多克隆、交叉克隆甚至返回原发部位（自接种）。Psaila等^[31]^提出转移前壁龛（premetastatic niche）理论，认为原发肿瘤会释放一系列信号分子，使转移灶周围的成纤维细胞活化，募集骨髓中的造血干细胞以及循环免疫抑制细胞，从而改变局部微环境，且所有这些变化都是在肿瘤细胞到达转移位点前完成的。

转移分化时间是指转移克隆首次存在的时间，而不是细胞从原发部位迁移的时间。早期分化是指原发肿瘤中有一组特定基因突变在转移灶中完全不存在，表明在转移分化后原发肿瘤内发生了完整的克隆清除（PE模型单克隆转移灶）。晚期分化是指患者原发肿瘤和所有转移灶中都有同一组基因突变，表明在转移分化后原发肿瘤内没有额外的克隆清除（BE模型多克隆转移灶）^[32]^。

全基因组测序（whole genome sequencing, WGS）来自两个未配对的原发性和转移性队列的7108对71种癌症的肿瘤样本和匹配的正常样本基因组^[33]^。在23种癌症类型中，与原发肿瘤相比转移性肿瘤每例患者的点突变[包括SBS、双碱基置换（doublet-base substitution, DBS）和INDELs]只有适度的增加，其中的15种癌症类型中的任何突变类型的突变负担都没有显著增加。大多数癌症类型的平均增加幅度低于每个样本1.5个驱动基因突变，并且所有突变类型（扩增、缺失和突变）都导致了转移性肿瘤中驱动基因突变的增加。内源性（例如SBS1和APOBEC）和外源性致癌过程（例如铂类药物治疗可操作变异）存在高度可变的肿瘤特异性。转移性病灶通常具有较低的ITH，染色体臂非整倍体显示了一个普遍保守的图谱。与肿瘤突变负荷（tumor mutational burden, TMB）相比，SV分析显示了更广泛的泛癌效应，每种转移性癌症类型都有较大的增加，几乎影响了所有研究的癌症类型。

发生转移的患者常见于年轻男性，原发性肿瘤富含微乳头状或实体组织学亚型，并具更高的TMB、CIN和基因组倍增率。TP53、SMARCA4和CDKN2A的失活与特定部位转移时间的缩短相关。APOBEC突变特征在转移瘤中更为普遍，尤其是肝脏病变。对匹配标本的分析^[34]^表明，原发性肿瘤和转移瘤之间通常共享相同的致癌和可操作变异，但意义不明的基因CNV往往是转移瘤特有的。只有4%的转移灶具有在其匹配的原发灶中未检测到的用于治疗的可操作变异。

### 7.4 基因检测和液体活检

肿瘤分子进化是具有时间和空间差异的动态过程。从临床诊断看，单次采样ITH对BE和NE模型影响显著，而对LE和PE模型影响有限。因此目前对肿瘤的取材和测序方式越来越倾向于多点取材、纵向监测的模式。近来对循环肿瘤细胞（circulating tumor cells, CTCs）和循环肿瘤DNA（circulating tumor DNA, ctDNA）或者患者衍生异种移植物（patient-derived xenograft, PDX）的研究也许能够解决这一问题，同时影像学的发展也是解决这个问题的途径之一^[35]^。

### 7.5 治疗策略

尽管吸烟是肺癌最重要的危险因素之一，但仍有大约25%的肺癌患者一生中吸烟少于100支。与吸烟者肺癌相比，不吸烟者肺癌表现出独特的基因组结构，包括较低的TMB，这些遗传改变的普遍存在使不吸烟者肺癌的靶向治疗获益更多。然而，不吸烟者肺癌对PD-L1免疫治疗的反应要差得多，提示不吸烟者肺癌的TME可能与吸烟者肺癌不同，因为TME在免疫治疗中起着重要作用^[36]^。

依据分子进化模型推导出肺癌进化存在长树干、树枝很少的特点，提示肺癌内部亚克隆之间的共性大、差异小、药物靶向治疗的效果较好，但是靶向药物治疗的耐药问题一直困扰着临床。在治疗开始之前，耐药突变（原发耐药）可能存在于肿瘤群体中，通常是次要的亚克隆；如果它们以非常低的频率存在或局限于肿瘤的未采样区域，则可能会逃避基线样本中的检测；在药物最大耐受剂量治疗的选择性压力下，对治疗敏感的人群减少，使耐药人群在正选择下展开PE模型，可以选择适应性治疗的必要性最小剂量策略。治疗耐药性可能是一种新突变的结果（继发耐药），这种突变在治疗中具有选择优势，并在肿瘤群体中固定下来，因此，耐药需要更长的时间才能出现。还有一种进化可以使肿瘤获得对其他药物的敏感突变，建议选择序贯治疗。

总之，肺癌发生发展的遗传多样性选择假说体现在肺癌发生、MPLC起源、原发灶和转移灶的分子进化过程，并为基因病理检查的时空性和药物治疗的策略提供了理论基础。

**Competing interests**

The author declare that there was no competing interests.
